# Optimised conformal total body irradiation: a heterogeneous practice, so where next?

**DOI:** 10.1259/bjr.20220650

**Published:** 2023-01-14

**Authors:** Sarah Misson-Yates, Rissa Cunningham, Regina Gonzalez, Patricia Diez, Catharine H Clark

**Affiliations:** 1 Department of Medical Physics, Guy’s Cancer Centre, Guy’s and St Thomas’ NHS Foundation Trust, London, UK; 2 School of Biomedical Engineering & Imaging Sciences, King's College London, London, United Kingdom; 3 Radiotherapy Physics, National Radiotherapy Trials Quality Assurance Group (RTTQA), Mount Vernon Cancer Centre, Northwood, UK; 4 Metrology for Medical Physics Centre, National Physical Laboratory, Teddington, UK; 5 Radiotherapy Physics, University College London Hospitals NHS Foundation Trust, London, UK; 6 Medical Physics and Bioengineering Department, University College London, London, UK

## Abstract

The use of volumetric arc therapy and inverse planning has been in routine use in radiotherapy for two decades. However, use in total body irradiation (TBI) has been more recent and few guidelines exist as to how to plan or verify. This has led to heterogeneous approaches. The goal of this review is to provide an overview of current advanced planning and dosimetry verification protocols used in optimised conformal TBI as a basis for investigating the need for greater standardisation in TBI.

## Introduction

Total body irradiation (TBI) is a well-established external beam radiotherapy treatment employed as part of the conditioning regimen prior to bone marrow, haematopoietic or peripheral blood progenitor stem cell transplantation with the aim to supress the patient’s immune system and prevent rejection of the donor’s stem cells.^1,2^ High dose, myeloablative TBI treatments should also eradicate malignant tumour cells, particularly in sanctuary sites which chemotherapy may find more difficult to penetrate, such as the brain or the testes. Patients undergoing myeloablative TBI are at risk of significant toxicities, in particular to the lungs with the risk of developing interstitial pneumonitis..^2,3^ Patients who are unable to tolerate myeloablative TBI undergo lower dose single fractionation regimes (reduced conditioning or non-myeloablative TBI).^2^.

Radiation treatment consists of the delivery of high megavoltage (MV) energy photons to the entire body. Conventional TBI is usually delivered using a linear accelerator at an extended source-to-surface distance (SSD) so that the treatment field may encompass the entire patient, whether in a standing, lying or semi-seated position. At least two opposing fields are delivered, *e.g.* using a right- and left lateral for a semi-seated position. To achieve dose homogeneity within ± 10% (as specified in the American Society for Radiation Oncology (ASTRO) practice guideline^4^) throughout the patient, compensators are used. These consist of either lead or Perspex shielding, water equivalent bolus and/or multileaf collimators within the treatment field. *In vivo* dosimetry is recommended for TBI to verify the delivery of the prescription dose and doses to organs at risk (OARs).^5^ Methods to optimise 3D dose distributions and minimise doses to OARs could confer benefits to these patients and in particular paediatric cases.^2^

Optimised conformal TBI techniques are now being implemented in centres worldwide and there has been an upsurge in publications, particularly for the use of isocentric-based techniques such as volumetric arc therapy (VMAT)^6–9^ and helical tomotherapy (HT).^10–14^ These techniques bring many challenges due to the long treatment volume >140 cm, including planning protocol development, imaging verification, dose rate considerations and dosimetric verification of the treatment plan, which is critical to ensure accurate delivery. Elongated treatment fields require multiple arcs and this leads to overlapping junction regions where the dose needs to be carefully controlled. Modern treatment planning systems (TPSs) offer ways to limit the junction dose, either through the planning method or during optimisation of the plan. Junction regions need to be verified dosimetrically prior to treatment, however, this can be done alongside the standard patient-specific quality assurance (PSQA) performed for VMAT plans.

In paediatric TBI, decreasing doses to OARs is of paramount importance to reduce acute toxicity and late effects. The Children’s Oncology Group (COG) in the United States conducted a recent survey^15^ in which 79% of respondents supported investigation of new techniques to lower lung doses and 83% supported the use of VMAT or HT. The European SIOPE survey in 2021^16^ concluded that modern TBI techniques with integrated shielding would allow for the reduction of OAR doses. Recent guidelines for paediatric TBI^2^ have concluded that myeloablative TBI performance for children is currently heterogeneous and centre-specific.

## Introducing advanced techniques

Previous reviews of TBI practice support evaluation of optimised conformal techniques.^17–19^ Ideally, the introduction of advanced techniques should form part of a clinical trial, as stated in the International Lymphoma Radiation Oncology Group (ILROG) 2018 guidelines.^1^ Wong et al^20^ published a detailed review of total marrow irradiation (TMI) and total nodal irradiation (TNI) covering clinical trial results, review of technologies used for treatment (*e.g.* VMAT) and considered the barriers for implementation of VMAT and HT for TMI/TNI. They suggested that, to increase the uptake of TMI/TNI techniques, the first step should be to introduce intensity modulated radiotherapy (IMRT) for TBI and consider using artificial intelligence (AI) for contouring. Recent papers on VMAT TBI have also considered the use of planning automation^21,22^ and flattening filter free delivery^23^ to aid implementation of optimised conformal techniques.

Recent national TBI surveys across Europe, Canada, Japan, New Zealand, and the United States^24–28^ demonstrated that the majority of centres are still utilising an extended SSD technique with some use of field-in-field (FIF) techniques to improve dose homogeneity. These surveys highlighted multiple areas of heterogeneous practice, in particular the wide range of dose rates in use (2.25–40 cGy/min) and lung tolerance doses (8–12 Gy). Prescription points were only specified in 2/7 surveys and location varied from the umbilicus to an average of midline points. The use of dose–volume histogram (DVH) constraints in CT planning were only discussed in half the surveys. This provides an incomplete picture of the planning protocols in use.

If optimised conformal techniques are implemented as part of a clinical trial, accurate dosimetry is vital to report trial outcomes.^15^ External dosimetry audits are an important part of safety culture in radiotherapy.^29–34^ They are recommended as best practice for new equipment and techniques, and ensure dosimetric accuracy is maintained and improved. They provide assurance of accuracy for clinical trial participation and support implementation for optimised conformal techniques. The requirement for audit is specified in various recommendations including the NHS England External Beam Radiotherapy Specifications 2019 (NHSE EBRT 2019).^35^ TBI dosimetry audits are uncommon and, at the time of writing, there are no full papers published describing dosimetry audits on TBI in the literature. The Imaging and Radiation Oncology Core (IROC) Houston QA centre (Houston, Texas) have performed a paediatric TBI audit using a custom-made phantom simulating a 10-year-old. The audit was conducted remotely at 20 institutions to propose credentialing criteria. This confirmed the wide variety of TBI practices and lung doses in use.^36^

Recently published guidelines covering planning and delivery of TBI from ASTRO,^3^ ILROG,^1^ the Australian College of Physical Scientists and| Engineers in Medicine (ACPSEM)^37^ and the Netherlands Commission on Radiation Dosimetry (NCS)^38^, however, do not include complete guidance on planning protocols, volume definition, OAR dose-volume constraints and PSQA.

The development of TBI practice is rapidly evolving to incorporate optimised conformal planning techniques. Implementation is taking place independently within individual institutions, which has led to heterogeneous approaches in treatment planning and delivery. The aim of this review is, therefore, to evaluate the published planning and dosimetry protocols used for optimised conformal TBI to support standardisation of TBI practice.

## Current status of planning techniques

A literature search was conducted on the 23 April 2022 using PUBMED and the criteria included were total body irradiation AND planning within the last 10 years (2013–2022) due to the upsurge in publications of optimised TBI. This returned 364 papers, which were distilled down to 88 papers that referred to TBI planning. Papers had to contain details of the TBI planning protocol, including planning target volume (PTV) and OAR dose-volume constraints/objectives, to be included. Papers describing clinical results and protocols for an optimised conformal TBI technique as part of a planning study were also included. Conference abstracts, simulation planning studies with no clinical data or papers covering TMI only were excluded. 28 papers remained, which were also examined for details of PSQA and/or *in vivo* dosimetry measurements performed as part of clinical practice.

**Figure 1.**
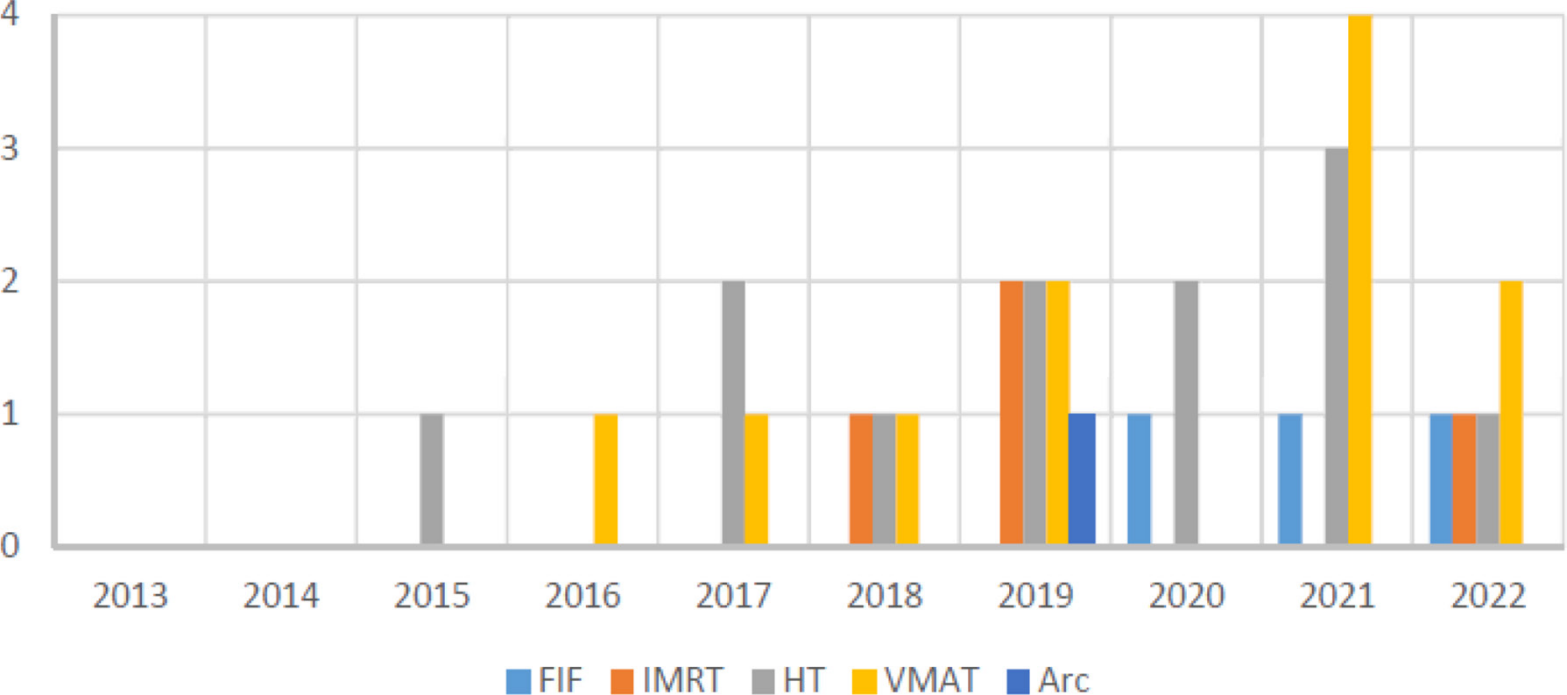
Frequency of papers published by technique.

These papers covered a range of techniques, as shown in Table 1. Four papers compared two TBI techniques giving a total of 32 protocols. HT (including TomoDirect)^10–14,39–45^ is the most widely used technique followed closely by VMAT^6–9,46,47^ or VMAT hybrid techniques, where the lower limb fields are delivered using a FIF anteroposterior/posteroanterior (AP/PA) technique.^48–51^ Extended SSD treatments such as FIF, IMRT^52–55^ and arc or modulated arc therapy^56,57^ also feature. Arc techniques require the patient to be placed on a specially designed couch near to the treatment floor with a supine then prone delivery of multiple arcs. Only one institution delivered VMAT using this setup.^48^ Comparing the techniques against year of publication it is evident that papers on VMAT implementation are on the rise (Figure 1). 13/32 of the protocols included paediatric patients, 3 were paediatric only. Modulated approaches were used in all cases for both paediatric and adult patients.

**Table 1.** Frequency of techniques used

Technique	Number of protocols
**Extended SSD FIF**	3
**Extended SSD IMRT**	3
**Arcs and/or static at floor**	1
**Modulated arc**	1
**VMAT near floor**	1
**VMAT and FIF**	2
**VMAT**	9
**HT**	12

FIF, field-in-field; HT, helical tomotherapy; IMRT, intensity modulated radiotherapy; SSD, source-to-surface distance; VMAT, volumetric arc therapy.

The findings have been summarised according to the ESTRO RATING^58^ methodology for reporting treatment planning results and protocols. Areas of common practice, used in more than half the protocols, were limited but included the use of head-first supine (HFS) and feet-first supine (FFS) CT scans due to limited scan length, the use of 6 MV and defining lung as a critical OAR.

## CT simulation

For the majority of VMAT and HT techniques, patients had full body CT scans with typical slice thickness of 5 mm, two scans (HFS and FFS), then either fused or planned as two separate plans (16/32). Alternatively, patients were scanned to mid-thigh supine and the dataset extended from the most inferior slice to represent the patient’s full height (2/32) or scanned with bent legs or to mid-thigh (7/32). Arms were placed along the patients’ sides in the majority of protocols (24/32). One paper referred to full body scanning but it is unclear how these were conducted, and four papers did not state the scanning method. When immobilisation techniques are mentioned, vacbags are the main equipment employed (7/32) to ensure setup consistency during HFS and FFS rotation, as well as thermoplastic masks around head and shoulders.

## PTV definition

PTV definitions differ. Over two-thirds of the protocols (21/32) based the PTV definition on the whole-body contour with a 3–5 mm margin cropped away from the skin to account for modelling inaccuracies and to avoid overdosing at the skin surface, whereas 5/32 reported the whole body for the volume definition. The inclusion or exclusion of OARs from the final PTV was variable with only half the protocols (16/32) excluding them. The OARs excluded also varied making it difficult to compare PTV dose–volume statistics directly. The most widely used PTV dose-volume constraint was D95 >95% followed by inspection of dose coverage between 80 and 95% of the prescribed dose. These parameters are, of course, directly influenced by the PTV definition. PTV definition and plan prescription for external beam radiotherapy also need to follow standardised protocols to allow comparison of plan dosimetry (ICRU 83^59^).

## PTV coverage/dose–volume objectives

Planning PTV objectives vary widely across protocols. Approximately, half the protocols set hard constraints and either used dose–volume parameters or a visual inspection of dose coverage. The most frequently used dose–volume objectives are given in Table 2, with D95 >95% of the PTV being the most common.

**Table 2.** PTV coverage and dose-volume objectives

PTV dose objectives	Protocols
D95 > 95%	8
Coverage > 95%	2
Coverage > 90%	6
Coverage > 85%	1
Coverage > 80%	2
D98 90%	3

PTV, planning target volume.

## Organs at risk

### Lung

The lungs are critical OARs due to the risk of developing interstitial pneumonitis (IPS) at around 8–10 Gy,^1^ except from low dose conditioning indications. The main dose constraint reported was mean lung dose (MLD), ranging from 7 to 12.5 Gy, again confirming a lack of consensus. Seven protocols used a MLD constraint <10 Gy, nine a MLD of 10–12.5 Gy, and one reported a range of 8–10 Gy, depending on prescription dose. Other lung dose constraints used included V10Gy <10%,^43^ V10Gy <40%,^40^ V8Gy >90% with D_median_ <9 Gy^42^ and V6Gy >90%, V8Gy <40%.^55^ MLD >8 Gy has also been associated with a decrease in overall survival^60^ and recent paediatric TBI guidelines recommend limiting MLD to <8 Gy.^2^

### Kidneys

Kidneys were the second most prevalent OAR with dose-volume constraints stated in 10 of the papers, with a mean dose limit to combined kidneys between 7.5 and 13 Gy. For paedatric TBI a mean dose <10 Gy is recommended.^2^

### Other OARs

TBI treatments are also known to cause toxicity to organs other than the lungs and kidneys, including the brain, eyes, (with example constraints D_mean_ < 12 Gy^54^ and D_max_ <4–6 Gy, respectively^6,8^) and liver. For paediatric TBI, reducing the lens dose to <12 Gy is recommended.^2^

## Long-term risks

In the paediatric setting, the increased ability to conform treatments could have benefits for reducing other important long-term effects such as secondary malignancies, infertility, cardiovascular disease, learning deficits and growth failure.^18,60–62^ In recent years, the radiosensitivity of the heart has become an increasing area of research, to predict risks of cardiovascular disease.^63^ The risk of premature death following haemopoietic stem cell transplant (HSCT) carries a 2.3-fold increased risk due to cardiovascular complications compared to healthy individuals (including TBI).^64^ Cardiac doses can be reduced even when using conventional shielding, as highlighted in a recent paper by Glenn et al,^65^ although 3D optimisation could take this further. Paix et al^18^ state that HT for TMI *vs* conventional TBI provides a mean dose reduction of between 46 and 47%. Even with these dose reductions, optimised conformal TBI included in HSCT regimes needs to be compared to HSCT regimes using conventional TBI delivery techniques to assess their true effectiveness in terms of dose-toxicity and outcomes and, ideally, as part of a clinical trial.^20^

## Treatment machines, TPS and energy

The treatment machines used for delivery were stated in 23/32 protocols. HT was the most frequently used machine (10/32) with the Siemens linear accelerator the least frequent. Consequently, the most commonly used TPS was HT (14/32). For VMAT and IMRT-based techniques, Eclipse and Pinnacle were the most widely used TPSs (9/32 and 7/32 respectively). For VMAT techniques, the number of isocentres ranged from 3 to 15 and the number of arcs planned at each isocentre varied between 2 and 8 arcs. Energies were stated in approximately half of the protocols, with 6 MV the most frequently used (range 6–18 MV).

Where dose rates were stated, these were reported in MU/min or cGy/min in relation to the beam or patient, respectively. Reported dose rates ranged from 0.1 to 19 cGy/min for extended SSD techniques or 20 MU/min to 600 MU/min for VMAT/HT techniques. Absorbed dose rates were reported at a range of locations, when specified, including mid-plane and in lung. In some cases, the mean instantaneous dose rate was reported.

Extended SSD TBI techniques, tended to report a dose rate of 300 MU/min with one (step and shoot IMRT) reporting 100 MU/min and another (FIF+VMAT) where the legs were irradiated with 600 MU/min fields.

7/32 protocols using modulated VMAT techniques only reported the average dose rate, while 6/32 protocols made the distinction of lower dose rates being used on arcs irradiating the lungs (*i.e.* 40 or 60 MU/min for adults and 20 MU/min for children). The rest used 300 MU/min, on average.

Dose rate was not usually reported for HT techniques. Instead, average beam-on time or modulation factor and/or pitch were discussed: The longer the treatment time (within reason for patient’s comfort) the lower the dose rate, and the more homogenous the plans.

For VMAT/HT, 3D dose rate mapping from the TPS could be advantageous as this would enable future analysis of planning dosimetry data *vs* toxicity data.

Dose rate is often cited as a risk factor for IPS^3,19^ and, hence, moving to isocentric techniques, where dose rates are much higher, could increase IPS incidence. Vogel et al^3^ published a critical review of pulmonary toxicity after TBI and recommended that a dose rate <15 cGy/min and a MLD <600 cGy was supported by the literature. This conflicts with other papers that recommend a MLD<8 Gy. However, this detailed review of over 100 papers not only found inconsistencies in definitions of IPS but also in the reporting of TBI parameters limiting detailed research into their correlation. Nevertheless, with incomplete reporting of TBI parameters and the lack of clarity on dose rate definition further dosimetric evaluation will not be possible until TBI reporting has reached a sufficient level of standardisation.^2^ Only then will it be possible to undertake a comprehensive assessment comparing lung dose–volume statistics, dose rate and IPS incidence and severity.

## Fractionation

**Figure 2.**
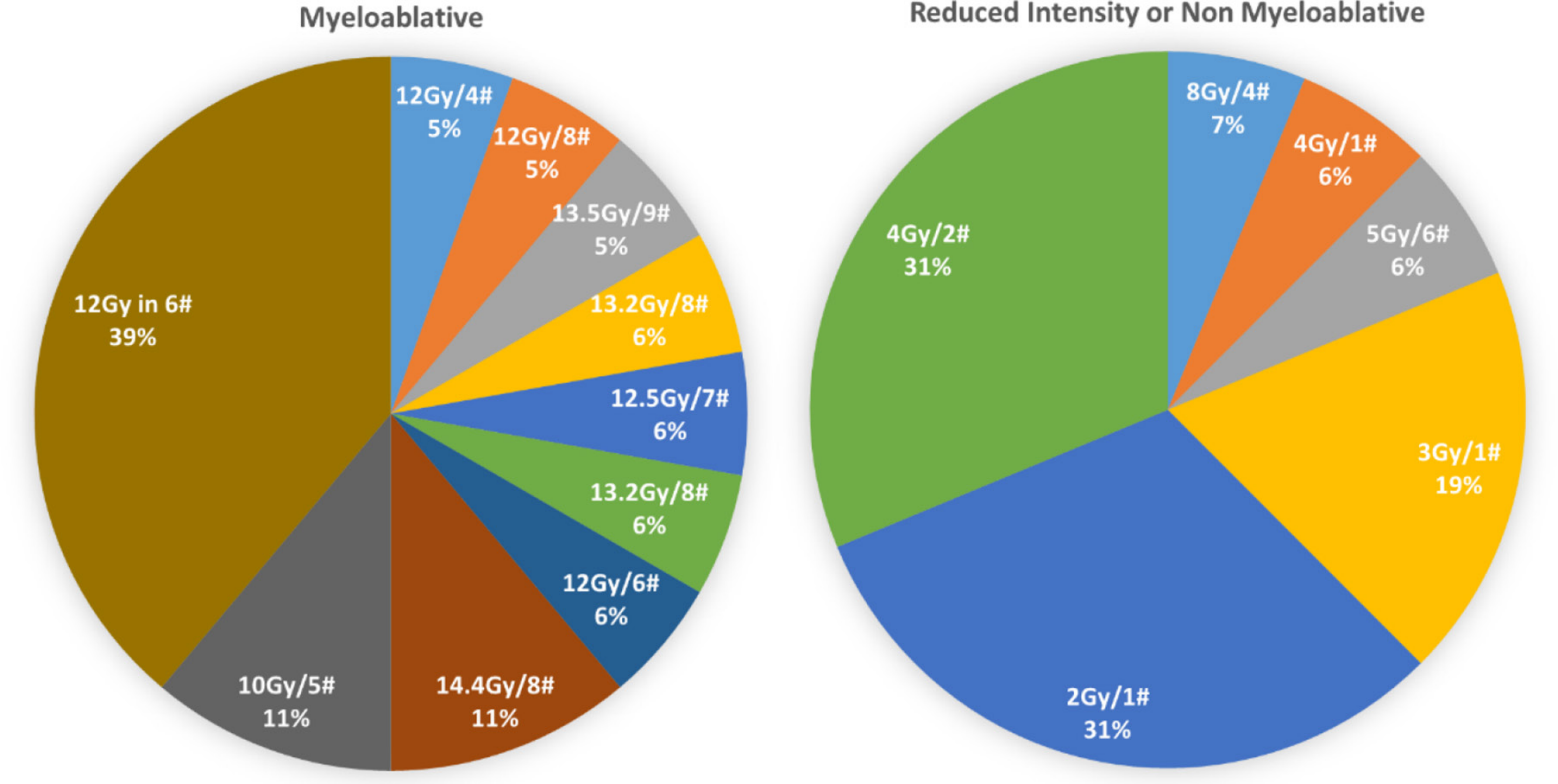
Frequency of fractionations for myeloablative or non-myeloblative and reduced intensity regimes.

A wide range of fractionations were used as shown in Figure 2. 11 different fractionations were used across 24 centres. The most frequently used fractionation was 12 Gy in 6 fractions, reported in 5 publications. The most commonly used dose per fraction (#) was 2 Gy, reported by over half of the papers and ranging from 0.83 to 3 Gy. Fractionation, and therefore prescribed doses, varied widely resulting with total doses up to 12–14.4 Gy for high-dose regimes.

## Junction dosimetry

**Figure 3.**
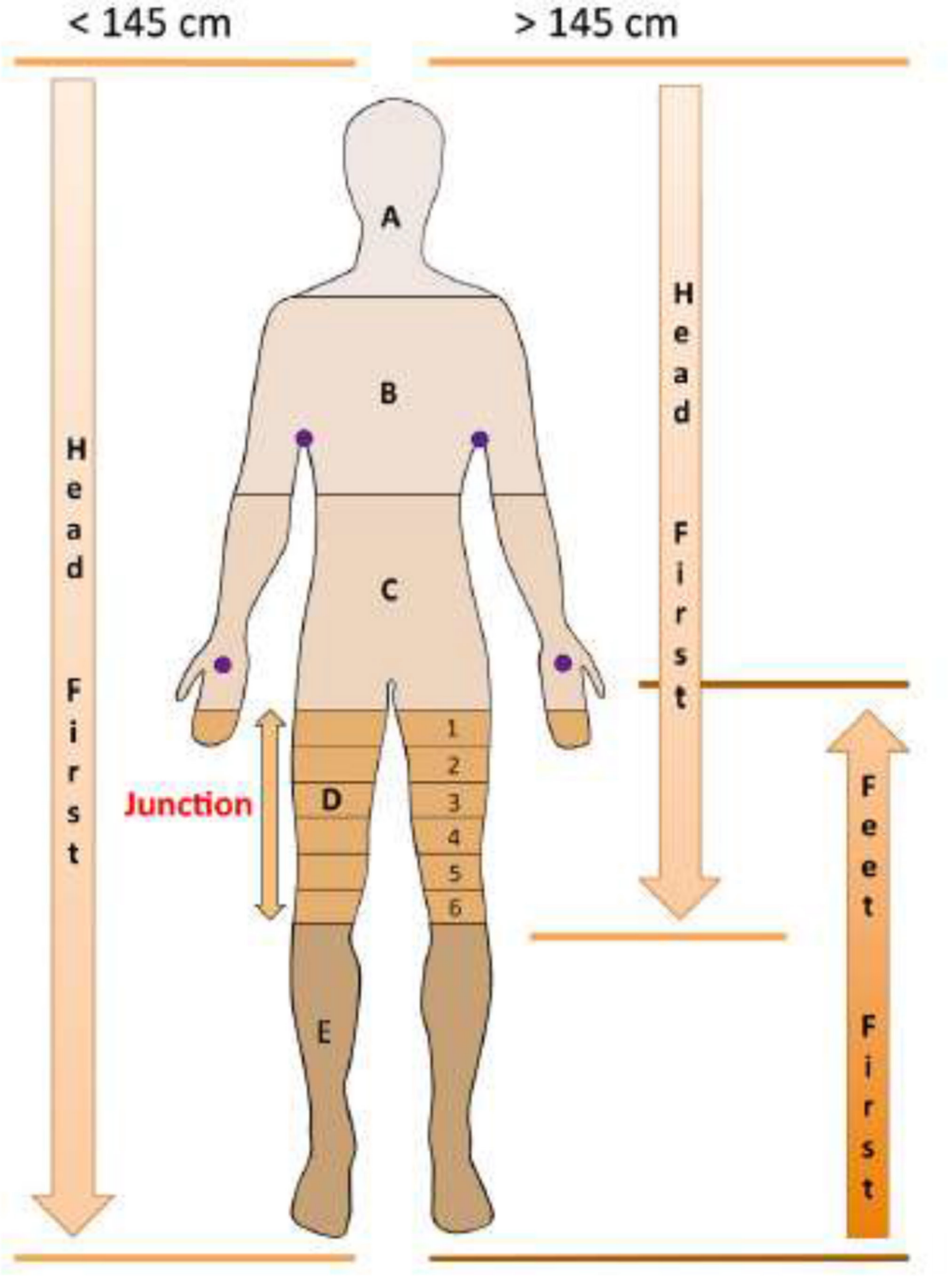
PTV subvolumes (reproduced with permission^11^)

Optimised conformal TBI planning comes with practical challenges, particularly in delivering a uniform dose to a PTV > 140 cm whilst minimising junction doses. VMAT and HT full body TBI isocentric techniques require optimisation of the junctions between arcs or superior and inferior plans. There is variation in how institutions handle this region. Of the 21 relevant papers, 17 presented details of how dosimetry was approached (Table 3). For VMAT planning, the majority of centres used either an additional beam or overlap between arcs which the TPS would use to account for the dose distribution during optimisation. For HT, the approach was distinctly different, with PTV subvolumes being created in the overlap region, usually the mid-thigh, with different optimisation objectives to create a dose gradient in opposite directions for the superior and inferior plans. This meant that, on summation, a uniform junction dose could be achieved. Figure 3 shows an example of the subvolumes.^11^ Only one institution used PTV subvolumes for their VMAT technique.

**Table 3.** Junction optimisation for VMAT and HT plans

Junction optimisation method	Technique	Number of Protocols
PTV subvolumes	HT (3), VMAT (1)	4
PTV amendment to allow for junction volumes	HT	1
Manual adjustment	HT	3
TPS optimised with arc overlap	VMAT	5
TPS FIF MLC optimisation	VMAT	1
TPS optimised using isodose structure	VMAT	1
TPS optimised additional beam	VMAT	2

FIF, field-in-field; HT, helical tomotherapy; MLC, multileaf collimator; PTV, planning target volume; TPS, treatment planning system; VMAT, volumetric arc therapy.

Recent guidelines for paediatric TBI state that field junctions should be evaluated as part of departmental QA procedures and planned dose homogeneity within the target should be within 90–110% of the prescription dose. Few centres stated their acceptable junction dose range (3/17; 14%). There was also minimal reporting on measurement and verification of the junction region for PSQA^44,46^ (2/17).

## Patient-specific quality assurance (PSQA) and *in vivo* dosimetry

**Figure 4.**
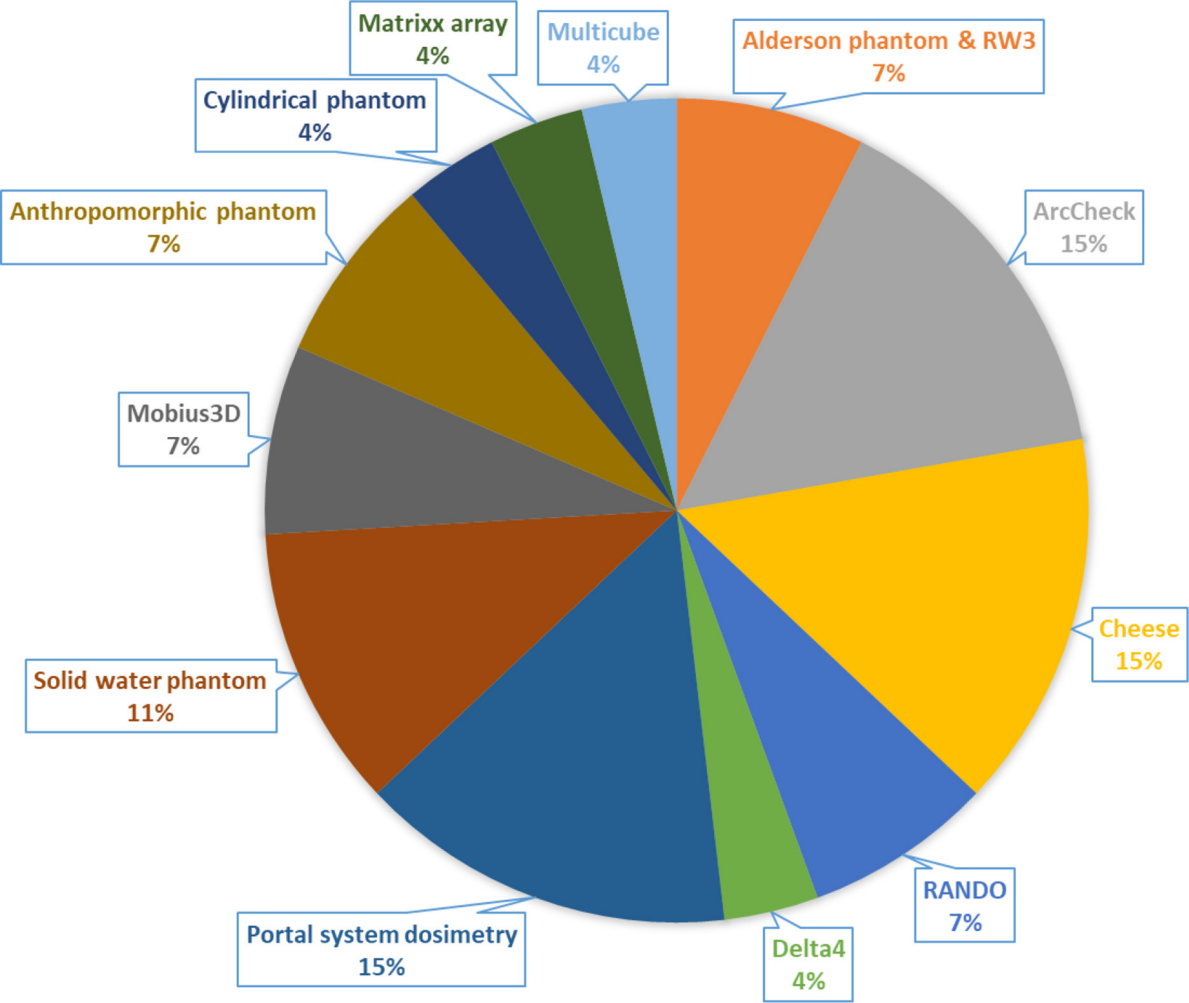
Phantoms used for PSQA

The final aspect to consider for implementation of optimised conformal techniques includes verification of the treatment plan delivery. A large number of methods and equipment were reported to verify TBI treatments, however, from the papers analysed, only half commented on how PSQA was performed. The majority (11/19) of centres took an absolute dose measurement to verify the dose from the TPS, with other methods including array or *in vivo* measurements. The remainder did not report any PSQA or *in vivo* measurements. Several phantoms were used across the centres including solid water, TomoTherapy “Cheese phantom” and Alderson in combination with an ionisation chamber (NACP parallel plate chamber, A1SL), (Figure 4). Other methods used for PSQA included a 2D or 3D array such as ArcCheck, Delta4 and Multicube, where gamma analysis was performed to evaluate the dose distribution. Of nine centres using phantom based PSQA, six did not state the pass rate criteria for gamma analysis. However, the remaining three centres used a 3%/3 mm gamma pass rate criterium with two of the centres passing the plan over 90% and the last centre using a stricter criterium of over 95%. It was not stated how the gamma pass rate was decided, or any other criteria used.^66^ According to the recommendations in AAPM TG218,^67^ the suitable gamma criteria to assess a standard VMAT plan is 3%/3 mm, but the guidance does not specify any recommendations for TBI treatments.

Finally, some centres aimed to confirm delivered dose *in vivo* using TLDs, OSLD, MOSFET and portal system dosimetry. The majority of the *in vivo* dosimeters were positioned in the thorax and abdomen (but varied in position between centres) with a maximum tolerance of 10% compared to the calculated dose. Within the study comparison, half of the centres (13/33) did not report their method for verifying the dose and several papers stated more than one method of PSQA and/or *in vivo* dosimetry. *In vivo* dosimetry using TLD, OSLD or MOSFET is typically performed on the skin. The body surface dose can be non-uniform in VMAT techniques thus reducing the accuracy of *in vivo* dosimetry. This may be one reason why centres choose not to perform *in vivo* dosimetry.

With a move to more optimised conformal TBI techniques with complex deliveries, recommendations for appropriate methods of verification and suitable tolerances become increasingly important. A final safety check should also include an external, independent dosimetry audit.

## Conclusion

Optimised conformal TBI techniques offer the ability to reduce doses to OARs, which could lead to a reduction in toxicity. These advanced techniques, combined with organ and target delineation, will also allow for reporting on a wider range of plan dosimetry parameters enabling more in-depth research into correlation with clinical outcomes and toxicity. However, in order to do so and for these techniques to be appropriately incorporated into future clinical trials, there must be standardisation and collaboration within the international TBI community. The key finding of this review is the limited yet varied extent to which TBI planning, delivery and dosimetry protocols are reported in the literature. This alone demonstrates the need for standardisation, *e.g.* following the ESTRO RATINGS methodology^58^ for reporting radiotherapy planning techniques. Consensus guidance is required for PTV outlining, setting planning objectives and OAR dose-volume constraints and recommendations provided on verification and dosimetric audit of these complex techniques to confirm delivery accuracy. Only then can we fully evaluate and research the potential benefits of these optimised conformal methods for TBI treatment.
